# Six Letters with Replies

**Published:** 1876-10

**Authors:** 


### Our Correspondence.

NATCHEZ, Miss., Sept. 1st, 1876.

DR. UP DE GRAFF :

Is there any way of correcting a slight squint in the eye of a young girl, save by operation?

J. B. S., M. D.

Yes. It can be accomplished by adjusting prisms, or convex lenses to the eyes, depending upon the *cause* of the squint. Operation is the quickest and most satisfactory, when admissible.

---

MONTROSE, Pa., Oct. 18, 1876.

DR. UP DE GRAFF :

I see you have an advertisement of Warner's Pills in your journal. How comes it? I suppose they are "patent medicines" and therefore unworthy our attention.

J. D. B., M. D.

Not at all. Warner's Sugar Coated Pills are prepared from prescriptions furnished by the profession, and are put up in this convenient form especially for the use of the physician. They are not secret nostrums, but reliable formulary employed by the regular practitioner.

---

The following brief letter explains itself and shows that the Philadelphians are disposed to be moderate in their charges for the board and lodging of their centennial visitors :

PHILADELPHIA, June 26, 1876.

*Dear Doctor :*—I would like to state that there is a wide spread impression that hotel and board charges are extraordinarily high, and your last No. of the BISTOURY refers to it. Board is not higher than at ordinary times. First-class private board at $2 per day, and medium as low as $6 to $8 per week, and even as low as $5 for very respectable board. A glance at our advertising ledger will show this.

Yours, A. S. L.

---

SYRACUSE, July 14, 1876.

DR. UP DE GRAFF :

Will you answer the following queries in your next issue, "answers to correspondents?" Can you recommend any particular kind of "shoulder brace" to straighten the figure of a growing girl? and could you fit or arrange any thing of that kind by having the bust and waist measures sent you, without seeing the subject?

My daughter is thirteen, and not very strong. She had a fit of sickness some years ago, and upon her recovery I found she had leaned to one side so much, she had become somewhat one-sided, one shoulder being higher than the other. Her chest is narrow, and a tendency to stoop over and thrust out her neck is becoming more manifest. Under these circumstances I would like your advice. Respectfully,

MRS. E. K. W.

You had better have your daughter's spine examined. She *may* have a lateral curvature—very common occurrence for one of her age. It would be a fatal mistake to neglect such a trouble. If a spinal curvative exists, have a gypsum jacket applied immediately, and by one capable of applying it properly.

---

SUNBURY, Pa., Aug. 19th, 1876.

MY DEAR DOCTOR :

I like your sharp BISTOURY. Send it as long as I can see to read. I am now 80 years old. Send me, if you please, the common name of *Xanthium Spinosum.* What is it, where can I procure it? C. F. S., Sr.

Its common name is *cocklebur.* Grows on sandy shores and waste places. The branching stems of the plant are armed with slender, triple prickles at the base of the narrow, short-petioled leaves. Bur small, with slender beak-like tip.

---

SYRACUSE, N. Y., Aug. 5th, 1876.

DR. UP DE GRAFF :

As soon as the BISTOURY reached our city it was astonishing to witness the dropping off in the patronage of "The Great English Physician," whose humbugery you so richly exposed. You can scarcely find a man that will acknowledge that he patronized the scamp, while they all "knew him to be a rogue," of course. After the exposure he packed his traps and departed for parts unknown, but he will have a hard time getting out of the reach of the BISTOURY, will he not?

B.

If your citizens will learn to distrust all traveling doctors, they will have taken a step in the right direction. Remember, always, that if these "doctors" can accomplish the cures they claim for themselves, there could exist no possible excuse for their traveling about the country. All honest, successful physicians find enough to do at home. You will never find them perambulating about the country, stopping for a few days in a place, seeking patronage by means of handbills, a display of anatomical preparations, and declaiming from the writings of others, from the platform and calling them lectures.

It is always safe never to trust a traveling doctor.

